# Supplementary material for: Promoting Healthy Aging for Older People Living with Chronic Disease by Implementing Community Health Programs: A Randomized Controlled Feasibility Study
Source: Int J Environ Res Public Health. 2024 Dec 13;21(12):1667. doi: 10.3390/ijerph21121667 (PMC11675327; doi:10.3390/ijerph21121667)
Supplement: Supplementary file 1 [file ijerph-21-01667-s001.zip › Supplementary file S1.pdf]

Supplementary File S1

Table of Contents

Document	Page
Participant survey questions	2
Staff survey questions	4
Secondary outcome measures	5
Participant activity attendance	7
Consort checklist	8

Participant survey CONNECT 50+ study.

PART 1: DATE:

1. Why did you decide to join the study?
2. What are your thoughts on the workbook? *(ie did you read it/ refer to it/ too much information/ too little/ completing the diary)*
3. Were you able to use the FitBit? *(was it easy/ difficult to set up/ monitor/ retrieve the step count/ assistance from Laolu?)*
4. Were there any issues/ problems with Laolu contacting you weekly for your step count?
5. Do you feel the Step count monitoring influenced your activity levels? If so, how?

NB: Questions 7-20 are for intervention participants only

6. What have you learnt from the program?
7. Which physical activity programs did you choose to attend?
8. Do you feel you have increased your level of physical activity over the course of the program? *(ie did the program encourage you to do more physical activity in general?)*
9. Which Wellness activity did you choose to attend?
   What did you think?
10. Which activities (either Physical Activity or Wellness activities) did you enjoy the most and why?
11. Which did you least enjoy and why?
12. Were there any other activities you were interested in attending? *(if so, which ones?)*
13. Do you feel you have made meaningful social connections throughout the program? *(NB if participant chose an "active" type wellness activity, ask if they think it may have been different or may have enhanced social interaction if one of the more social type activities was accessed eg book club/ craft etc)*
14. Has this changed anything about how you connect socially outside of the program? *(eg increased confidence, joined other groups, attended other events eg lunches)*
15. Have you had to overcome any barriers to participate in the program? *(eg not interested in programs, transport difficulties, health issues)*
16. What made it easier to participate in the program? (eg welcome at desk, variety of programs, good instructors).
17. Would you recommend the program to a friend?
    Why/ Why not?

18. What changes to the program would you suggest?
19. a) Are you interested in continuing for a further 3 months? (**at completion of 3 months initial program**) Why/ why not?
20. Which programs specifically?

NB: Question 21 is for control participants only

21. Now that you have completed the 3 months of monitoring only, are you interested in joining CONNECT for a 3-month period. *(discuss type of program eg – physical activity/ wellness)*

PART 2: DATE:

22. Did you sign up for the bonus 3 month free CONNECT membership?
    (Why/ why not?)
23. Which classes did you attend/ how often?
24. Are you likely to continue to attend programs at CONNECT (on a fee-paying basis?)
    (Why/ why not?)

Staff participant survey CONNECT 50+ study.

Research staff:

1. What was your role in the CONNECT 50+ study?
2. Describe the facilities at CONNECT:
3. How effective was the recruitment process? (*issues accessing potential participants, difficulty contacting participants, any changes you would make)*
4. Discuss baseline measures: (*booking, time frame, challenges eg room availability, any changes you would make)*
5. Fitbit set up and monitoring: (c*hallenges eg medical conditions, smart phone/ email, participant understanding, weekly monitoring)*
6. Programs: (a*ny comments on programs offered/ attended by participants)*
7. Describe your experience in the study: (w*hat did you learn from it)*
8. What would you change about the program?

Community Hub staff:

1. What was your role in the CONNECT 50+ study?
2. Describe the facilities at CONNECT:
3. Describe the program participants joined: *(physical/ wellness classes, general description of class, engagement/ compliance/ attendance)*
4. Describe your experience in the study:

Secondary outcomes measured at baseline and 12 weeks:

- Functional mobility measured using the Timed Up and Go test [1,2]. The TUG measures the time a person takes to stand from a seated position in a chair, walk three metres at a comfortable pace, turn, walk back to the chair, and sit down. Normal mobility in healthy older adults is indicated by a time of 11 seconds or less [3].
- Gait Speed [4], measured as the time taken to walk three metres at a comfortable speed on a level surface. Each participant undertook two trials, and the quickest of the two times was recorded.
- Perceived wellness, measured by the Perceived Wellness Survey [5]. This is a 36-item, self-report measure of an individual's perceived wellness across six dimensions (physical, spiritual, psychological, social, emotional, and intellectual). It is scored on a Likert scale from 1 (Very Strongly Agree) to 6 (Very Strongly Disagree), with a Wellness Composite Score achieved by dividing the Wellness Magnitude (sum of the six subscale means) by the Wellness Balance [(square root of the variance) + 1.25].
- Health-related Quality of Life was assessed using the Health Questionnaire (EQ5D-5L; EuroQol) [6], a self-report measure of current health. It is comprised of five dimensions (mobility, self-care, usual activities, pain/ discomfort and anxiety/depression), with participants indicating their health status across five levels from 'no problems' to 'extreme problems'.
- Participants also rated their "health today" on the Visual Analogue Scale (EQ-VAS) [6] which has end points of 0 (worst health) to 100 (best health).
- Physical Activity (steps per day) [7] was measured daily by all participants using a smart watch (Fitbit) or pedometer and recorded on a weekly basis via telephone contact with Project Officer.

References

1. Podsiadlo D, Richardson S. The timed "Up & Go": a test of basic functional mobility for frail elderly persons. *J Am Geriatr Soc,* 1991;39(2):142-8. doi: 10.1111/j.1532-5415.1991.tb01616.x
2. Shumway-Cook A, Brauer S, Woollacott M. Predicting the probability for falls in community-dwelling older adults using the Timed Up & Go test. *Phys Ther Rehabil*, 2000;80(9):896-903. doi: 10.1093/ptj/80.9.896
3. Bohannon, RW. Reference values for the timed up and go test: a descriptive meta-analysis. *J Geriatr Phys Ther,* 2006;29(2):64-68. doi:10.1519/00139143-200608000-00004
4. Middleton A, Fritz S, Lusardi M. Walking speed: The functional vital sign. *J Aging Phys Act*, 2015;23:314-322. doi: 10.1123/japa.2013-0236
5. Adams T, Bezner J, Steinhardt M. The conceptualization and measurement of perceived wellness: integrating balance across and within dimensions. *Am J Health Promot,* 1997;11(3):208-218. doi:10.4278/0890-1171-11.3.208
6. The EuroQol Group. EuroQol-a new facility for the measurement of health-related quality of life. *Health Policy*, 1990;16(3):199-208. doi.org/10.1016/0168-8510(90)90421-9
7. Wu S, Li G, Du L, Chen S, Zhang X, He Q. The effectiveness of wearable activity trackers for increasing physical activity and reducing sedentary time in older adults: A systematic review and meta-analysis. *Digit Health*, 2023;9. doi:10.1177/20552076231176705

Participant Activity Attendance

Type of Activity	Specific group	Participant ID							
		**a**	**b**	**c**	**d**	**e***	**f**	**g**	**h**
**Strength and Balance**	Active for life (Circuit)	9	0	0	0	0	3	0	1
	Active for Life	11	5	3	0	0	1	7	8
	Feel Your Best Fitness	0	2	0	0	0	1	0	6
	Zumba	0	0	0	5	0	0	0	0
	Pilates	0	0	0	4	0	0	3	10
	**Total**	**20**	**7**	**3**	**9**	**0**	**5**	**10**	**25**
**Wellness/ Social**	Chair Yoga	0	5	2	2	0	0	0	1
	Shibashi Tai Chi	12	0	0	0	0	0	8	0
	Chapters (Writers Group)	0	0	0	0	0	0	5	0
	Community Lunch	0	0	4	0	0	0	0	0
	Independence and Wellness	0	0	1	0	0	0	0	0
	**Total**	**12**	**5**	**7**	**2**	**0**	**0**	**13**	**1**
	**Grand Total**	**32**	**12**	**10**	**11**	**0**	**5**	**23**	**26**
	**Ave weekly attendance**	2.7	1.0	0.8	0.9	0	0.4	1.9	2.2
* participant returned to full-time work									

CONSORT 2010 checklist of information to include when reporting a pilot or feasibility trial[a]

Section/Topic	Item No	Checklist item	Reported on page No
**Title and abstract**			
	1a	Identification as a pilot or feasibility randomised trial in the title	1
	1b	Structured summary of pilot trial design, methods, results, and conclusions (for specific guidance see CONSORT abstract extension for pilot trials)	3
**Introduction**			
Background and objectives	2a	Scientific background and explanation of rationale for future definitive trial, and reasons for randomised pilot trial	4
	2b	Specific objectives or research questions for pilot trial	6
**Methods**			
Trial design	3a	Description of pilot trial design (such as parallel, factorial) including allocation ratio	6
	3b	Important changes to methods after pilot trial commencement (such as eligibility criteria), with reasons	Figure 1 and page 13 (mentioning reduced sample size)
Participants	4a	Eligibility criteria for participants	7
	4b	Settings and locations where the data were collected	7
	4c	How participants were identified and consented	7,8
Interventions	5	The interventions for each group with sufficient details to allow replication, including how and when they were actually administered	8, 9

Outcomes	6a	Completely defined prespecified assessments or measurements to address each pilot trial objective specified in 2b, including how and when they were assessed	9,10. And supplement ary file 1.
	6b	Any changes to pilot trial assessments or measurements after the pilot trial commenced, with reasons	12
	6c	If applicable, prespecified criteria used to judge whether, or how, to proceed with future definitive trial	9, 27
Sample size	7a	Rationale for numbers in the pilot trial	12
	7b	When applicable, explanation of any interim analyses and stopping guidelines	n/a
Randomisation:			
Sequence generation	8a	Method used to generate the random allocation sequence	8
	8b	Type of randomisation(s); details of any restriction (such as blocking and block size)	8
Allocation concealment mechanism	9	Mechanism used to implement the random allocation sequence (such as sequentially numbered containers), describing any steps taken to conceal the sequence until interventions were assigned	8
Implementation	10	Who generated the random allocation sequence, who enrolled participants, and who assigned participants to interventions	8
Blinding	11a	If done, who was blinded after assignment to interventions (for example, participants, care providers, those assessing outcomes) and how	8
	11b	If relevant, description of the similarity of interventions	9 see control group conditions
Statistical methods	12	Methods used to address each pilot trial objective whether qualitative or quantitative	11,12
**Results**			
Participant flow (a diagram is strongly recommended)	13a	For each group, the numbers of participants who were approached and/or assessed for eligibility, randomly assigned, received intended treatment, and were assessed for each objective	Figure 1
	13b	For each group, losses and exclusions after randomisation, together with reasons	Figure 1
Recruitment	14a	Dates defining the periods of recruitment and follow-up	6

	14b	Why the pilot trial ended or was stopped	27
Baseline data	15	A table showing baseline demographic and clinical characteristics for each group	Table 1
Numbers analysed	16	For each objective, number of participants (denominator) included in each analysis. If relevant, these numbers should be by randomised group	Table 3
Outcomes and estimation	17	For each objective, results including expressions of uncertainty (such as 95% confidence interval) for any estimates. If relevant, these results should be by randomised group	Table 3
Ancillary analyses	18	Results of any other analyses performed that could be used to inform the future definitive trial	Results feasibility sections
Harms	19	All important harms or unintended effects in each group (for specific guidance see CONSORT for harms)	n/a
	19a	If relevant, other important unintended consequences	n/a
**Discussion**			
Limitations	20	Pilot trial limitations, addressing sources of potential bias and remaining uncertainty about feasibility	Section 3.2.6 expansion and page 30 limitations
Generalisability	21	Generalisability (applicability) of pilot trial methods and findings to future definitive trial and other studies	30
Interpretation	22	Interpretation consistent with pilot trial objectives and findings, balancing potential benefits and harms, and considering other relevant evidence	27-29
	22a	Implications for progression from pilot to future definitive trial, including any proposed amendments	27-29
**Other information**			
Registration	23	Registration number for pilot trial and name of trial registry	6
Protocol	24	Where the pilot trial protocol can be accessed, if available	n/a, details of protocol in trial registry

Funding	25	Sources of funding and other support (such as supply of drugs), role of funders	31
	26	Ethical approval or approval by research review committee, confirmed with reference number	32
a = Eldridge SM, Chan CL, Campbell MJ, Bond CM, Hopewell S, Thabane L, et al. CONSORT 2010 statement: extension to randomised pilot and feasibility trials. BMJ. 2016;355.			
