# Supplementary material for: Promoting Healthy Aging for Older People Living with Chronic Disease by Implementing Community Health Programs: A Randomized Controlled Feasibility Study
Source: Int J Environ Res Public Health. 2024 Dec 13;21(12):1667. doi: 10.3390/ijerph21121667 (PMC11675327; doi:10.3390/ijerph21121667)
Supplement: Supplementary file 1 [file ijerph-21-01667-s001.zip › Supplementary file S2.pdf]

CONNECT 50+ Healthy ageing for midlife and beyond

WORKBOOK

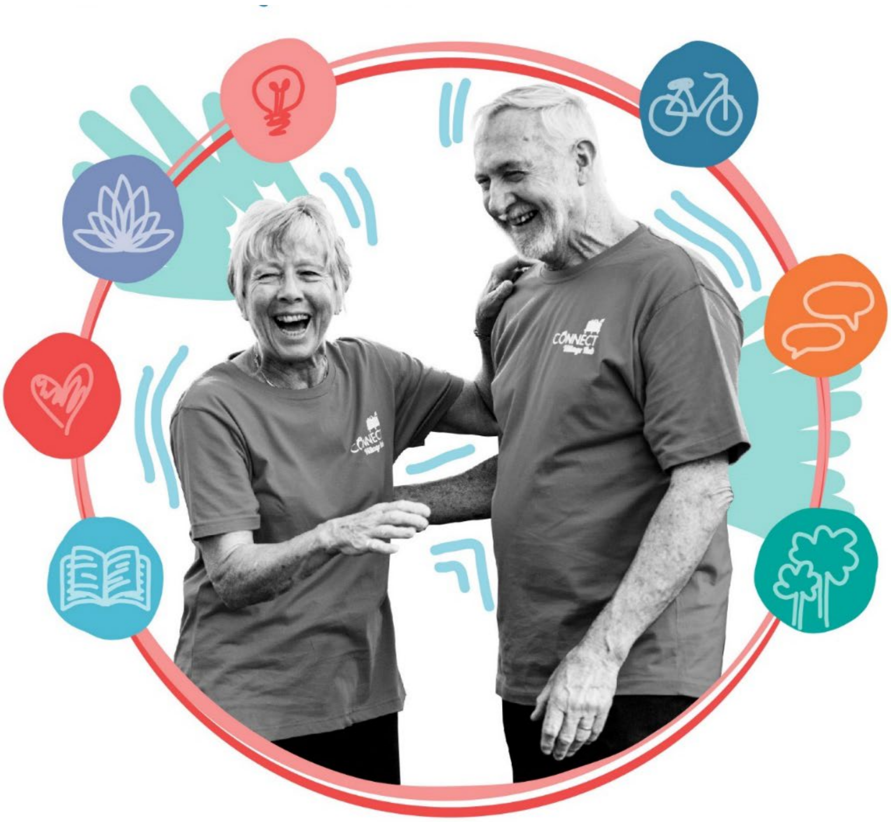

Welcome to Connect 50+

Healthy ageing for midlife and beyond

We acknowledge the traditional custodians of this land – the Whadjuk people of the Noongar nation – and pay respect to their past, present and emerging leaders, continuing cultural heritage, beliefs and relationship with the land.

Connect 50+ is brought to you by Connect Victoria Park Village Hub, University of Western Australia School of Allied Health, and WA Centre for Health and Ageing.

We are excited to embark on a **12-week wellness journey** with you exploring seven domains as recommended by the International Council on Active Ageing (ICAA).

- Physical
- Social
- Intellectual
- Emotional
- Environmental
- Vocational
- Spiritual

Connect 50+ acknowledges financial support received from RPH Research Foundation Springboard Plus Grant.

Program Overview

Connect 50+ Healthy ageing for midlife and beyond is an evidence-based 12-week healthy ageing program for wellness.

It is designed for people 50 and over who experience chronic diseases and want to keep engaged, maintain or increase quality of life, and live independently for longer in the communities they love.

The program explores the International Council on Active Ageing's **seven domains of wellness**.

Connect Victoria Park | Village Hub

Connect 50+ is an initiative of Connect Victoria Park Inc., a non-profit organisation that has provided **affordable housing** for older people for over 60 years.

We operate 74 units on Mackie and Cargill Street in Victoria Park and currently have 80 tenants.

In 2018, Connect Village Park launched the **Village Hub**, the first "virtual village" in Western Australia and the second in Australia.

Virtual villages like ours were created in the United States in 1990s by older people wanting to connect and support each other, keep active and increase their changes of living independently for longer.

Our Village Hub is a community of around **400 members** who like to do things together, be it exercise, volunteering or socialising.

We have a range of classes and courses available to everyone over 50 and members have access to Help Centre – tech savvy sessions, Justice of the Peace, help with filling forms and navigating the system – and Neighbour-to-Neighbour, a program that links people in need of help with small tasks at home with volunteers who can help.

Village Hub members enjoy **increased quality of life** and have found a community of friends to share their interests, skills and experience.

Connect 50+ is a great showcase for the Village Hub, as you will have a taste for several activities regularly offered. Feel free to take additional classes for the duration of the program and ask staff and members more about the Hub.

After Connect 50+, becoming a Village Hub member is a great option to deepen your wellness experience and continue the activities you found most beneficial.

We hope your Connect 50+ experience is invigorating and helps you live well now and in the future!

University Research

Connect 50+ is not only a program of classes and activities, it is also part of a formal **research study** by University of Western Australia.

The study will evaluate improvements in physical and functional ability because of engagement with the program, as well as benefits for health-related quality of life and social connectedness. The research will provide valuable evidence of how older people were enabled to socially connect and improve their physical ability and quality of life after recent hospitalisation.

Connect 50+ participants who choose to be part of the study will be interviewed face to face at Connect Village Hub with a trained research health professional at the start of the program and will have some basic health markers recorded. A similar interview will happen at the end of the 12-week program. Evidence gathered will be analysed and synthesized and it's expected to show the effects of physical, cognitive and intellectual stimulation, as well as emotional balance, for people experiencing chronic diseases as they age.

Connect 50+'s results will inform future programs at Connect Village Hub, as well as support scientific publications by the researchers, with potential to be translated into improved policy and practice across the public health system in Western Australia. Reporting and dissemination of findings internationally through research journals, will provide a valuable guide on how best to deliver a Wellness program and critical success factors.

University Human Research Ethics Committee (HREC) has approved this study. Please do not hesitate to contact Dr. Chiara Naseri at chiara.naseri@uwa.edu.au or 0409 570 448 if you have any questions about the study.

Why wellness?

Wellness is an active process through which people become aware of, and make choices toward, a more successful existence.

It's not uncommon for the media to equate wellness to physical activity. If we are interested in whole-person wellness outcomes, however, there is much more to it.

The International Council on Active Ageing (ICAA) names seven domains that contribute to wellness: **physical, emotional, spiritual, intellectual, social, environmental and vocational.**

The dimensions reflect different areas of a person's life, and balance is hard to achieve without some investment of time, attention and resources.

Especially as we age, wellness is particularly important – at the individual level, but also for society as a whole.

Over the century, we have added an additional 30 years or so to life expectancy in developed countries. This single fact is the cause of a wave of change that impacts economies, businesses, governments, communities, families and individuals.

More people living longer means increased demand for health services, and therefore more costs to society, but it also means opportunity to explore the potential embedded in the added life expectancy. That's if people stay healthy and well as they age, keep participating and contributing to community, fully engaged with life.

Chronic diseases and disabilities that were once thought as inseparable from old age continue to join the ranks of those that can be prevented, or at least controlled, often through changes in lifestyle – diabetes type 2 being a classic example.

In this program, we separated wellness into 7 dimensions so we can offer avenues for you to start or continue exploring aspects of life that may enhance your wellbeing. On a day-to-day basis, however, the dimensions overlap and coordinate, and wellness becomes a valuable framework that serves wants-and-needs of a person engaged in life.

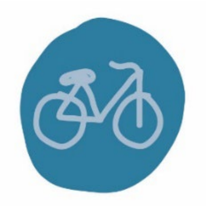

Physical Domain

Choosing lifestyle habits that maintain or improve health and functional ability.
Examples: exercise, fitness, nutrition, sleep, self-care.

International guidelines recommend that people should be physically active every day to help maintain fitness, strength, and ability to remain independent in everyday activities. Physical activity in daily life can be categorised into sports, household, active recreation and play, and can be done at any level of skill and for enjoyment by everybody. Regular physical activity is proven to help prevent and manage noncommunicable diseases such as heart disease, stroke, diabetes and several cancers. It also helps prevent hypertension, maintain healthy body weight and can improve mental health, quality of life and well-being. Exercise is a subset of physical activity that is planned , structured and repetitive and has the goal of improving or maintaining physical fitness (World Falls Guidelines, 2022)

The World Health Organisation (2022 Physical Activity factsheet) recommends that all adults should do at least 150-300 minutes of moderate intensity aerobic activity or at least 75-150 minutes of vigorous-intensity aerobic physical activity or a combination of both throughout the week. Also do muscle strengthening activities on 2 or more days per week. For adults over the age of 65, physical activities should emphasise function and balance and strength training at moderate or greater intensity on 3 or more days a week to prevent falls.

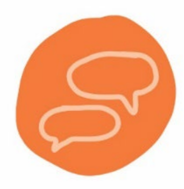

Social Domain

Socially connecting with others for mutual benefit, sense of self-worth and awareness of the larger community. **Examples:** clubs, volunteering, dancing, visiting friends and family, intergenerational activities, travel.

Relationships used to be much easier to form and maintain when people lived in smaller towns, knew and relied on their neighbours, and had family close by. Today, one quarter of Australians live alone, and security and safety concerns make people less inclined to connect with strangers, however according to the

World Health Organisation (Report on Ageing and Health, 2015), social connectedness is a key that enables people to have a better quality of life and age well, and have positive health benefits such as improved immunity and cognitive function.

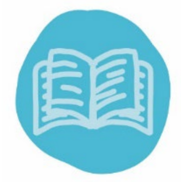

Intellectual Domain

Engaging in creative pursuits and intellectually stimulating activities, problem-solving and reasoning. **Examples:** cultural activities, arts and crafts, journaling, games/puzzles, reading, writing, learning new skills.

Trying something new and keep learning are great ways to keep your brain engaged and active.

We're bringing elder Roni Forrest for some storytelling so we can learn a bit about the Indigenous history of our area. Jacqui Masters, a local Victoria Park artist and teacher, will then lead us on a storytelling exercise so we can all have a go at imagining and relating a story.

Our brains, however, don't benefit only from purely intellectual and creative activities. It's known that exercise contributes to better memory and thinking skills and lower risk of dementia, but science is still investigating what type and how much exercise is optimal for brain health.

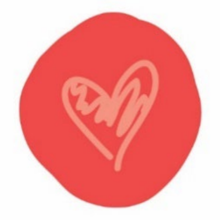

Emotional Domain

Managing and directing feelings; coping with challenges and behaving in trustworthy and respectful ways. **Examples:** peer support, humour, laughter, personal histories, celebrations, purpose.

We all know it can be hard to control feelings and emotions throughout life – but there are tools that make it is possible to gain some control and enjoy a calmer, more balance emotional life.

A very useful tool is meditation, which helps develop the ability to bring a heightened state of awareness to any activity, allowing us to slow down and enjoy the present moment.

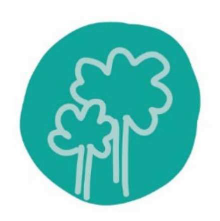

Environmental Domain

Immersing oneself in nature, observing animals, finding eco-friendly ways for cleaning, transport and daily activities, innovative processes and design. Examples: walking, hiking, camping, planting trees, gardening, repairing/mending/recycling.

As we age, we aim at being independent: to keep living in the same house, in the same neighbourhood, with the same community.

But interdependence – like in nature where the water cycle, soil and ocean health, air quality and biodiversity levels are intimately connected – is probably as important. No one will be completely independent throughout life, and especially as we get older we need more support and attention.

There is much to learn from nature, not to mention solace to be gained by being in nature.

We are fortunate in Vic Park to enjoy a remnant of pristine bushland, and all the life it contains, right in the middle of town. The Kensington Bushland is a Forever Site and part of the larger Jirdarup Precinct.

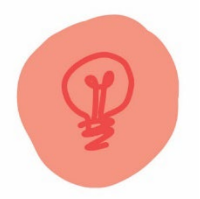

Vocational Domain

Maintaining, improving or transferring skills, abilities and attitudes that help self or others stay productive and satisfied with the work they produce. Examples: paid or volunteer work, skill share, mentoring, tutoring, hobbies, caregiving.

Wouldn't it be great if people were beating their way to your door for your expertise because you are experienced, older and wiser?

We may retire from work, but there is still plenty of worthy activities to dedicate ourselves to – especially after a lifetime of accumulating skills and experience.

Working part time, volunteering for a cause, caring for others or mentoring younger generations, older adults continue to be important contributors to society.

Spiritual Domain

Living with meaning and purpose in life, exploring beliefs and values that create personal peace and understanding. **Examples:** meditation, reflection, mindful exercise (tai chi, yoga), experiencing nature.

Spiritual wellness involves a set of guiding beliefs, principles or values that help give direction to one's life. Practicing mindfulness and/or faith-based observances and practices may help you manage stress and high blood pressure, sleep well, feel more balanced emotionally and ready to face your day.

A high level of commitment to your religious faith and/or individual beliefs provides a sense of meaning and purpose, and helps you find harmony between what lies within and the world outside.

Mindful exercise such as yoga is extremely helpful in developing those qualities, as it requires concentration, calming the mind and enhancing focus.

Mindfulness and meditation also help to identify the things in life that motivate and inspire you, the elements from within that you can offer the world, and those you require from outside in order to achieve your highest potential.

Physical and Wellness Activity Calendar

Monday	Tuesday	Wednesday	Thursday	Friday	Saturday	Sunday
